# Autotrophic nitrogen removal for decentralized treatment of ammonia-rich industrial textile wastewater: process assessment, stabilization and modelling

**DOI:** 10.1007/s11356-020-11231-y

**Published:** 2020-10-20

**Authors:** Simone Visigalli, Andrea Turolla, Giacomo Bellandi, Micol Bellucci, Elisa Clagnan, Lorenzo Brusetti, Mingsheng Jia, Roberto Di Cosmo, Glauco Menin, Martina Bargna, Giovanni Bergna, Roberto Canziani

**Affiliations:** 1 Department of Civil and Environmental engineering – Environmental section, Politecnico di Milano, Piazza Leonardo da Vinci 32, 20133 Milan, Italy; 2 AM-TEAM, Oktrooiplein 1, 9000 Ghent, Belgium; 3 Faculty of Science and Technology, Free University of Bolzano, Piazza Università 1, 39100 Bolzano, Italy; 4 Gruppo CAP, Via del Mulino 2, 20090 Assago, Italy; 5 Lariana Depur, Via Laghetto 1, 22073 Fino Mornasco, Italy

**Keywords:** Industrial wastewater, Decentralized treatment, Deammonification, Biological processes, PN/anammox process, Process scale-up

## Abstract

Digital textile printing (DTP) is a game-changer technology that is rapidly expanding worldwide. On the other hand, process wastewater is rich in ammoniacal and organic nitrogen, resulting in relevant issues for discharge into sewer system and treatment in centralized plants. The present research is focused on the assessment of the partial nitritation/anammox process in a single-stage granular sequencing batch reactor for on-site decentralized treatment. The technical feasibility of the process was assessed by treating wastewater from five DTP industries in a laboratory-scale reactor, in one case investigating long-term process stabilization. While experimental results indicated nitrogen removal efficiencies up to about 70%, complying with regulations on discharge in sewer system, these data were used as input for process modelling, whose successful parameter calibration was carried out. The model was applied to the simulation of two scenarios: (i) the current situation of a DTP company, in which wastewater is discharged into the sewer system and treated in a centralized plant, (ii) the modified situation in which on-site decentralized treatment for DTP wastewater is implemented. The second scenario resulted in significant improvements, including reduced energy consumption (− 15%), reduced greenhouse gases emission, elimination of external carbon source for completing denitrification at centralized WWTP and reduced sludge production (− 25%).

**Electronic supplementary material:**

The online version of this article (10.1007/s11356-020-11231-y) contains supplementary material, which is available to authorized users.

## Introduction

Digital (or ink-jet) textile printing (DTP) is rapidly spreading worldwide, mostly due to the greater versatility with respect to conventional printing techniques (Global Industry Analysts 2018). Despite lower wastewater volumes, discharges are rich in ammoniacal and organic nitrogen due to the massive use of urea as additive for pre-treating the fabric (Scaglione et al. 2016). Very often, such a high nitrogen content in process wastewater results in exceeding discharge limits for nitrogen in the sewer system (100 mgN/L in Italy), so that additional treatments are required, with consequent additional costs.

Over the last 20 years, the autotrophic removal of nitrogen by anaerobic ammonium oxidizing (anammox) bacteria emerged as a disruptive technology (Li et al. 2018). Anammox bacteria can be applied in synergistic combination with ammonium oxidizing bacteria (AOB) in the partial nitritation (PN)/anammox process, in which AOB oxidize about half of the ammonium present to nitrite (PN), while anammox bacteria use the nitrite produced and the residual ammonium to produce molecular nitrogen (Gonzalez-Martinez et al. 2018). Full-scale applications showed that the two biological reactions may take place either in two separate reactors or may be combined in one reactor thanks to the presence of both bacterial populations in consortia (Lackner et al. 2014; Gonzalez-Martinez et al. 2018). Such single-stage process, often based on granular biomass, showed excellent performance as sustainable alternative to established biological processes in terms of energy requirements (Hu et al. 2013). Moreover, among existing technological solutions, the use of a sequencing batch reactor (SBR) allows ensuring good biomass retention to favour the growth of anammox bacteria, avoiding their washing out, while the homogeneous distribution of substrates and products limits the formation of critical zones for bacteria survival inside the reactor (Lotti et al. 2014; Li et al. 2018).

Literature reported several full-scale experiences in which the PN/anammox process was successfully applied to industrial wastewater (Lackner et al. 2014), although the difficulties in achieving a stable process were equally strongly highlighted (Li et al. 2018), mostly represented by (i) the need for appropriate bCOD/N ratio (not exceeding about 2:1) and (ii) the inhibition of the biomass activity due to wastewater toxicity (Scaglione et al. 2016). In general, high bCOD:N ratios negatively affect the performance of PN/anammox processes, mainly due to existing competition between anammox bacteria and heterotrophic denitrifying bacteria. Indeed, under anoxic conditions, in terms of nitrogen removal efficiency, heterotrophic denitrification may become the most important metabolic pathway when biodegradable organic carbon is available (Gonzalez-Martinez et al. 2018; Cho et al. 2020). On the other hand, in order to favour PN reactions, it is necessary to prevent the activity of nitrite-oxidizing bacteria (NOB), which consume nitrite needed by anammox bacteria and produce nitrate. NOB can be inhibited by careful process management such as high temperature (higher than 30 °C), low specific retention time (SRT) (Jetten et al. 1998) and low dissolved oxygen concentration (0.25–0.5 mgO_2_/L) (Canziani et al. 2006; Jubany et al. 2009; Yoo et al. 1999). Similarly, to favour anammox species, temperature should be in the range 30–40 °C (Strous et al. 1997; Egli et al. 2001), while pH should be in the range 7–8 (Strous et al. 1999; Yang et al. 2006; Van Hulle et al. 2007; Carvajal-Arroyo et al. 2014; Puyol et al. 2014) since it regulates the concentration of free ammonia (high pH) and free nitrous acid (low pH), which are strong inhibitors at high concentrations. Moreover, anammox bacteria are favoured under anoxic conditions and even very low DO concentrations can reduce or inhibit their metabolic activity (Strous et al. 1997; Egli et al. 2001). A balanced combined PN/anammox process allows AOB, in the external layers of the granular biomass, to consume the oxygen present in the bulk, preventing it from coming into contact with anammox (Lotti et al. 2015).

Comparing the PN/anammox process with conventional nitrogen removal, substantial differences can be found. Nitrification involves large oxygen consumption, and consequently high energy consumption due to aeration, while denitrification requires large quantities of organic substance which acts as an electron donor for the reduction. In the PN/anammox process, oxygen consumption is significantly reduced as nitrification stops at nitritation and no organic substance is needed because both AOB and anammox are autotrophic bacteria. Furthermore, the autotrophic nature of these bacteria ensures low cell yield with reduced sludge production (van Dongen et al. 2001; Mulder 2003; Siegrist et al. 2008; Gonzalez-Silva et al. 2017; Cho et al. 2020). Such characteristics make the PN/anammox process a promising candidate for the above-introduced growing issue represented by DTP wastewater.

The EU’s LIFE DeNTreat project, whose experimental results at laboratory scale are reported in the present work, aims at demonstrating the feasibility of the PN/anammox process as a decentralized treatment for DTP wastewater. While such application has never been reported in literature to the best of authors’ knowledge, the main challenging aspects of the project are represented by the sub-optimal application conditions for the PN/anammox process and the need for a sustainable technological solution. In this research work, extensive experimental activity was carried out in a SBR laboratory pilot in the view of several goals: (i) assess the feasibility in reducing nitrogen concentration in compliance with regulations for discharge into the sewer system for wastewater from five DTP companies, (ii) identify optimal process stabilization condition, (iii) process modelling and (iv) techno-economic process assessment with respect to existing conventional treatment.

## Material and methods

### Wastewater characteristics and system layout

Most of experimental activities were carried out on the effluent of a DTP industry in the Como district (SCR - Stamperia di Cassina Rizzardi, Cassina Rizzardi, Italy). At present, nitrogen-rich wastewater is collected in a 1200-m^3^ equalization tank, from which it is discharged into the sewer system, thanks to a special authorization, and treated in a centralized wastewater treatment plant (WWTP). Such WWTP, designed for 24,000 population equivalent and treating more than 50% of industrial wastewater, is based on conventional activated sludge biological process. To comply with total nitrogen limit on WWTP effluent (15 mgN/L) by means of the biological process consisting in a raceway pre-denitrification tank and a completely stirred oxidation-nitrification tank, the addition of about 24 kgCOD/h, as a combination of glycerine, acetate and glycol, is required, by which a COD/N ratio of at least 6 is ensured.

Wastewater from SCR (WW 1) was collected about every 3 weeks from the equalization tank and constantly fed to the PN/anammox laboratory pilot for about 5 months. After tests on WW 1, some shorter tests have been carried out in the same reactor on samples of wastewater collected from four DTP companies: Satinskin S.A. (Mire de Tibães, Braga, Portugal), Estamparia Têxtil Adalberto Pinto da Silva S.A (Rebordões, Porto, Portugal), Stamperia di Lipomo (Lipomo, Como, Italy) and Stamperia Serica Italiana (Villa Guardia, Como, Italy), named as WW 2, 3, 4 and 5, respectively. WW 2 was collected downstream the viscose process, while WW 3, 4 and 5 consisted in the final effluent from treatment of different fabrics (i.e. cotton, silk and viscose). Among the four companies, only Stamperia Serica Italiana (WW 5) was equipped with an equalization tank. Wastewater characteristics are reported in Table 1.

**Table 1:** Wastewater characteristics: 6 samples for WW 1 (mean ± st.dev.), 1 sample for the others. Values for organic N, TKN, NH_4_-H/TKN and COD/TKN were estimated

Parameter	WW 1	WW 2	WW 3	WW 4	WW 5
pH	7.9 ± 0.3	8.9	8.8	9.2	9.2
Conductivity (μS/cm)	1001 ± 112	300	1537	2410	2430
TSS (mg/L)	187	70	33	NA	200
COD (mg/L)	690 ± 62	891	395	329	1001
TN (mg/L)	218 ± 29	728	508	220	311
NH_4_-N (mg/L)	168 ± 23	17	33	196	273
NO_3_-N (mg/L)	0.4 ± 0.6	1.3	4.9	0.8	2.7
NO_2_-N (mg/L)	0.0 ± 0.0	0.0	0.9	0.0	0.0
Organic N (mg/L)	49 ± 16	709	469	23	35
TKN (mg/L)	217 ± 29	727	502	219	308
NH_4_-N/TKN (%)	78 ± 6	2	7	89	89
COD/TKN	3.2 ± 0.5	1.2	0.8	1.5	3.2

In addition to wastewater from five DTP industries, a synthetic wastewater was prepared and used for reactor start-up and anammox biomass activity recovery. At the beginning of reactor start-up, it was used to dilute industrial wastewater to promote bacteria adaptation. The composition of the synthetic solution is described in Table S1 in Supplementary Material.

### Experimental setup

**Fig. 1:**
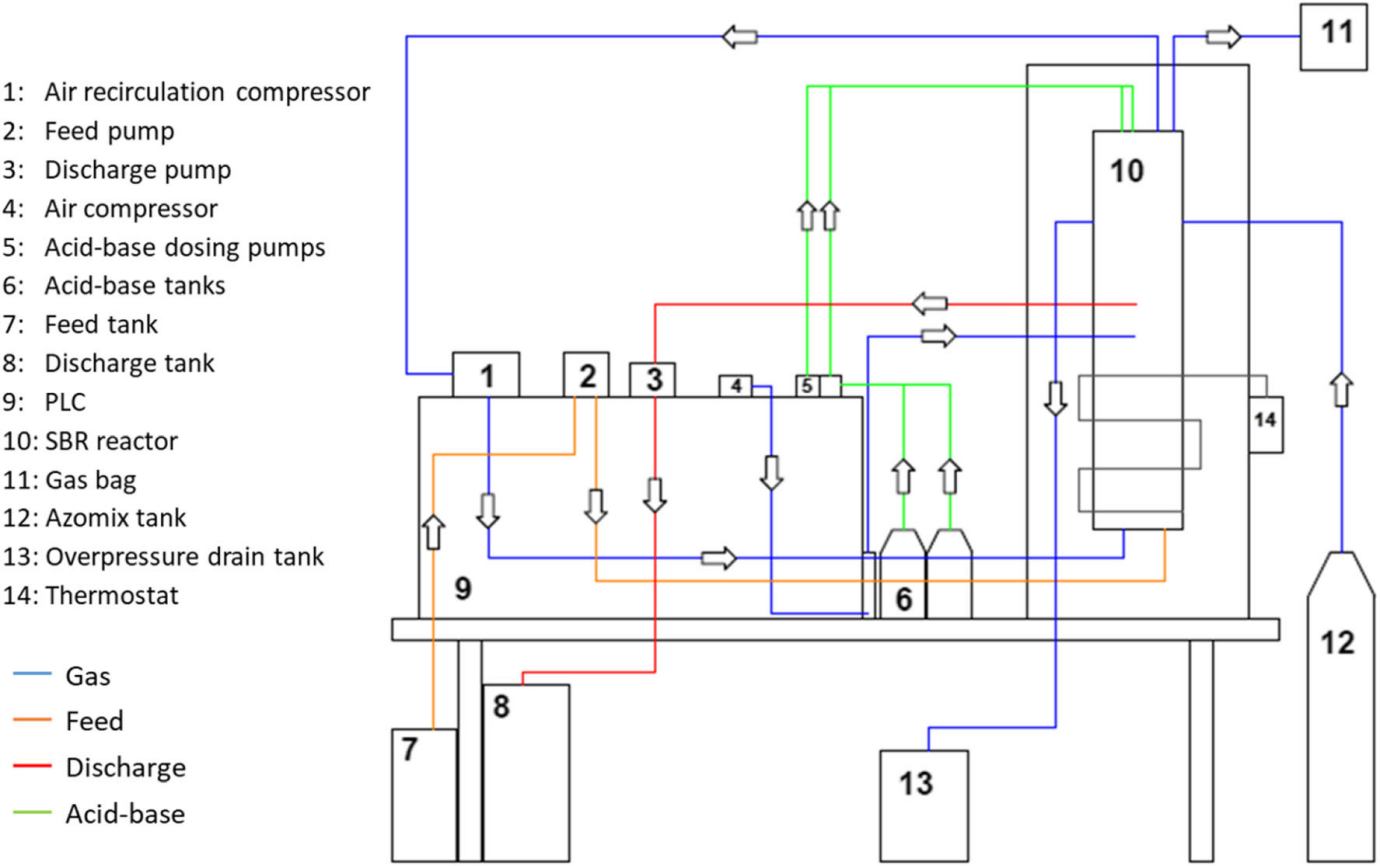
Layout of the PN/anammox laboratory pilot reactor

The PN/anammox reactor (Fig. 1) was a 2-L SBR that operated at about 32 °C in 2.5- to 6-h cyclic sequences comprising: (i) feeding phase, (ii) reaction phase, (iii) settling phase and (iv) discharge phase. During feeding and reaction phases, the reactor was mixed by recirculating and bubbling the gas in the head space in a closed loop. At steady state, the cyclic exchanged volume was 0.5 L. The process was controlled by PLC equipped with on-line sensors for temperature, conductivity, pH, oxidation reduction potential (ORP) and dissolved oxygen (DO). pH was controlled by HCl and NaOH dosage, while submerged aerators supplying ambient air were used for dissolving oxygen. Azomix E1 (Gruppo Sapio, Italy), a mixture of N_2_ and CO_2_ (~ 7%), was used during start-up to remove excess oxygen in the reactor, which could potentially inhibit anammox bacteria. The reactor was inoculated with two granular biomass batches from Paques (Balk, The Netherlands) at about 8 gVSS/L (VSS/TSS ratio = 0.75–0.80 gVSS/gTSS). The first batch (IN 1) was inoculated for the first 112 days of experimentation with WW 1, while the second batch (IN 2) was inoculated at the beginning of the second start-up period with WW 1 and it was also used for the short tests with WW 2, 3, 4 and 5.

### Experimental plan

The configuration of SBR cyclic sequences used in experimental activities is reported in Table S2 in Supplementary Material. The settling and discharge phases have always been maintained at 60 and 30 s respectively, to hinder the growth of suspended biomass. The duration of feed and reaction phases have been adjusted depending on the influent characteristics, the target nitrogen loading rate and the biomass activity. The experimental plan was structured in two main parts, respectively dedicated to treatment of WW 1 and wastewater from other DTP industries, as detailed.

#### WW 1

The experimental campaign on WW 1 was divided into two periods, a long-lasting set (112 days) and a shorter set (33 days) that were performed to assess the reactor stabilization. During the first start-up period, DO set-point values (between 0.2 and 0.8 mg/L) and cycle durations were progressively modified, while pH was always kept in the range 7.2–7.6. The laboratory scale reactor was initially fed with synthetic wastewater. Afterwards, the ratio between WW 1 and synthetic wastewater in the feeding was progressively increased (10–30–50%) until reaching undiluted conditions. Cycle duration was set at 3–4 h at the beginning of the experimentation, at low WW 1 ratios, and then increased to 6 h at undiluted conditions. After a period of 112 days, when stable conditions were achieved, the laboratory pilot was stopped for probe calibration and it was re-inoculated with a new biomass batch. The second start-up period was carried out under similar operating conditions, except for cycle duration, that was set at 4 h for the whole period. The reactor was restarted with synthetic solution for 3 days, followed by increasing of WW 1 ratios (25–60–100%) over approximately 30 days.

#### Wastewater from other DTP industries

Differently from tests with WW 1, experiments with wastewater from other DTP industries consisted in shorter tests that lasted around 5–10 days each. The aim of this activity was the evaluation of the treatability by the PN/anammox process, using optimal operating conditions emerged from WW 1 treatment. WW 2 and 3, due to the high initial total nitrogen (TN) concentrations, have been mixed in a 1:1 ratio with synthetic solution for 5 days and then tested at undiluted conditions. WW 4 and 5 were tested as collected, with no dilution. The pH set-point was kept in the range 7.2–7.6 for WW 2 and 3, while it was increased to 7.6–7.8 for WW 4 and 5. DO set-point was maintained at 0.2–0.4 mg/L for WW 2 and 3, while it was increased to 0.4–0.7 mg/L for WW 4 and 5. Cycle duration has been set according to the wastewater characteristics. It was kept constant at 6 h for WW 2, while values of 6 and 8 h have been tested for WW 3, in order to investigate the ammonification of organic nitrogen during the PN/anammox process. For WW 4 and 5, cycle duration has been initially set to 4 h and then reduced to 3 h.

### Analytical procedures

Commercial test kits, namely Hach Lange LCK 238, 303, 339, 342 and 514, were used respectively for TN, NH_4_-N, NO_3_-N, NO_2_-N and COD measurements on 0.45-μm filtered samples with a spectrophotometer LANGE Xion500. Total suspended solids (TSS) and volatile suspended solids (VSS) were measured according to the Standard Methods (Rice et al. 2017).

### DNA extraction and real-time polymerase chain reaction

Granular biomass (5 g of wet weight) of the two inocula and from the SBR was collected at days 18, 23, 35, 93 and 107 for the characterization of the N converting microbial community in the granules. DNA was extracted from each sample (500 μL, corresponding to about 0.577 g of wet and 0.011 g of dry biomass) in triplicate using DNeasy PowerSoil kit (QIAGEN) according to a modified version of the manufacturer protocol. The modification concerned the initial vortexing step, which was executed directly in the Eppendorf ThermoMixer Comfort at 1400 rpm for 10 min. The yield of the purified DNA was quantified using Qubit™ (Thermo Fisher Scientific) and extraction quality was controlled through gel electrophoresis 1% (w/v) 1×TAE agarose gel. DNA was stored at − 80 °C until quantitative polymerase chain reaction (qPCR) assays.

Prior to qPCR, extraction replicas were pulled together to reduce extraction variability. Real-time PCR amplifications were then performed using the Rotor-Gene SYBR Green PCR Kit (QIAGEN) according to the manufacturer’s instruction on a Rotor Gene real-time PCR cycler (QIAGEN). Each PCR reaction included an aliquot of 3 μL of DNA extracts, which were previously diluted 1:5 in nuclease-free water to reduce possible inhibition in a total volume of 15 μL of PCR master mix. PCR conditions and primer sets targeting gene encoding for the bacterial denitrification pathways (*nirS*, *nirK*, *nosZ*), bacterial ammonia monooxygenase (*amoA*) gene and anammox bacterial hydrazine oxidoreductase (*hzo*) gene are summarized in Table S3 in Supplementary Material.

Standard curves for absolute quantifications were created using specific DNA templates (from 10^8^ to 10^2^ copy numbers, 10-fold serial dilution series). These were synthetized by GeneArt Gene Synthesis (Thermo Fisher Scientific). The ordered standards were created by inserting the synthetic gene target sequence followed by a restriction site for PvuII within a vector plasmid. Standard plasmids were linearized with the restriction enzyme PvuII (Thermo Fisher Scientific), purified through PureLinkTM (Invitrogen – Life Technologies) and quantified though the use of Qubit® dsDNA HS Assay Kit (Molecular Probes– Life Technologies).

The absolute estimations of gene copies were converted into gene copies per gram of dried biomass, by determining the dry weight of each analysed biomass sample after 5 days at 60 °C.

### Process modelling and sustainability assessment

In addition to technical assessment of the PN/anammox process, the sustainability of the decentralized treatment was compared to the existing situation. Several Key Performance Indicators (KPI) for process impacts were identified, namely (i) energy consumption, (ii) greenhouse gas (GHGs) emissions, (iii) consumption of external carbon and (iv) sludge production. Data from DTP industry (SCR), centralized WWTP, wastewater characterization and experimental results from the PN/anammox reactor with WW 1 have been used for the modelling and assessment of two scenarios:

- Scenario 0 (business-as-usual): wastewater treatment by centralized WWTP, in which the DTP industry effluent is conveyed by the sewer system without any pre-treatment, as described in the “Wastewater characteristics and system layout” section. TN load to the WWTP is about 80,000 kgN/year.
- Scenario 1: decentralized treatment of WW 1 at DTP industry before discharge into the sewer system (around 1000 m^3^/day and 150–200 kgN/day, for a total of around 60,000 kgN/year) by means of the PN/anammox process, followed by treatment of wastewater conveyed by the sewer system in the centralized WWTP.

In both scenarios, the centralized WWTP was simulated by means of a modelling approach based on the work of Barker and Dold (1997) and developed by means of BioWin software (EnviroSim). The PN/anammox process was modelled using the same software and adapting the tool dedicated to sequential reactors with granular biomass (Granular Sludge Sequencing Tank, GSST). This tool, developed from the work of Takács et al. (2007), models granular sludge as a one-dimensional biofilm, while the sedimentation of suspended solids during the non-aerated and unmixed process phases is based on a flow model for one-dimensional solids.

The PN/anammox process modelling was based on experimental data from last period of experimental activity on WW 1, including different nitrogen species and COD components. Sizing and kinetic parameters are reported in Tables S4 and S5 in Supplementary Material. Default values provided by the software were initially assumed. Model calibration resulted in the adaptation of some kinetic parameters, as detailed in the following. For NOB bacteria, the specific growth rate has been reduced from 0.7 to 0.4 day^−1^ and the aerobic decay rate has been increased from 0.17 to 0.21 day^−1^ to hinder their growth and allow their washout. For anammox bacteria, the growth rate has been increased from 0.2 to 0.25 day^−1^ to encourage their activity. For ordinary heterotrophic organisms (OHO), the growth rate was reduced from 3.2 to 0.01 day^−1^ to hinder their predominance over other bacteria. In addition, during model setup, it was necessary to pay particular attention to the definition of parameters related to oxygen mass transfer and granule sedimentation rate.

KPI have been evaluated for the two scenarios using simulation results. Although not reported for brevity, a sensitivity analysis was carried out for these indicators.

## Results and discussion

### Preliminary process assessment

Wastewater characterization is reported in Table 1. For WW 1, the concentration of organic nitrogen (mostly composed by urea, as confirmed by company communications) was considerably lower than that of ammoniacal nitrogen. As already described, WW 1 was sampled from the equalization tank, collecting all waterflows downstream printing and washing processes. It is possible to assume that ureolysis phenomena occur in this tank, converting most of the organic nitrogen into ammoniacal nitrogen. A similar situation was observed for WW 5. On the other hand, the concentration of organic nitrogen was considerably higher than that of ammoniacal nitrogen in WW 2 and 3, being such observation possibly explained by the lack of an equalization tank in related DTP industries. Such a low concentration of ammoniacal nitrogen may suggest that WW 2 and 3 could not be treated by the PN/anammox process due to the impossibility for AOB species to convert ammonia to nitrite for anammox bacteria. Conversely, hydrolysis of urea was spontaneously promoted in WW 4, in which transformation yield was about 89%, although related DTP industry was not equipped with an equalization tank.

Previous respirometry tests evidenced that only a portion (usually 40–60%) of the COD of this industrial wastewater is biodegradable (bCOD) (Scaglione et al. 2016). Therefore, bCOD/TKN ratio lower than 2, which was indicated as favourable for anammox bacteria growth (Jin et al. 2012; Lotti et al. 2014), is satisfied for WW 2, 3 and 4, while the ratio is close to the limit for WW 1 and 5.

### Process stabilization

**Fig. 2:**
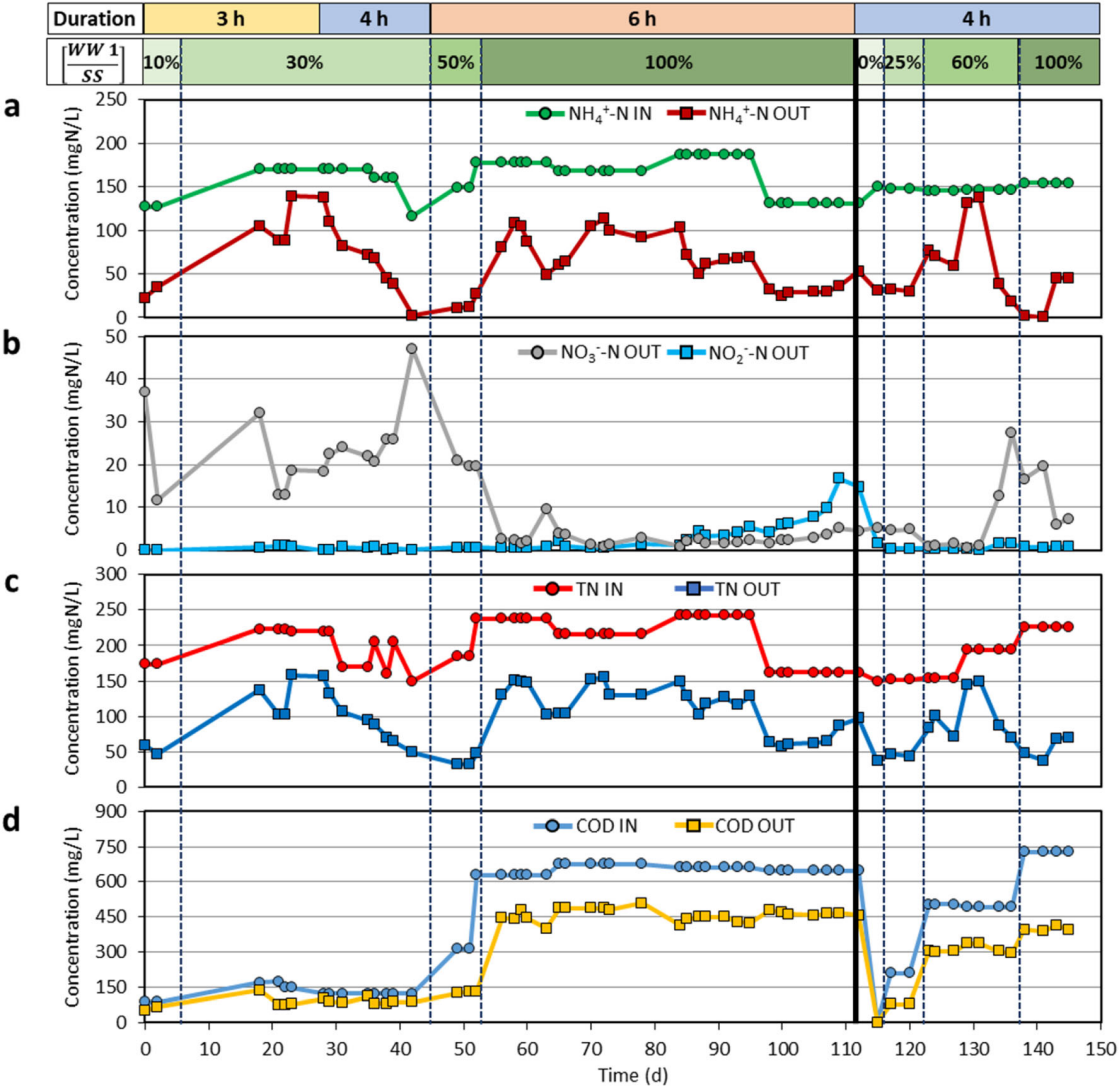
Composition of the PN/anammox reactor influent and effluent for experimental activity on WW 1 that was divided into two periods: **a** ammoniacal nitrogen (NH_4_-N), **b** nitrite (NO_2_-N) and nitrate (NO_3_-N), **c** TN and **d** COD. Cycle duration and wastewater dilution ratio are reported

Figure 2 shows the composition of the PN/anammox reactor influent and effluent, in terms of NH_4_-N, NO_2_-N, NO_3_-N, TN and COD, for experimental activity conducted on WW 1. The first start-up of the reactor confirmed the importance of an acclimation period for reaching steady-state conditions, being the progression in feed ratio between WW 1 and synthetic wastewater reported in Fig. 2. The anammox bacteria adapted over time, with nitrogen removal efficiencies increasing from day 20 to day 40. WW 1 was fed without dilution from day 50, as evidenced by the increase in COD concentration. Bacterial consortium was negatively affected by this modification in conditions, and the nitrogen efficiency sharply dropped from about 80% to less than 50%. It can be deduced that an abrupt change in the influent led to a reversible shock for bacteria that reduced their metabolic activity at day 55. Influent variability and the use of different colourants and additives might be the explanation for such observation. Indeed, biomass activity could have been affected by the presence of azo dyes, which cannot be removed by the biological process and could generate inhibiting metabolites and cause chronic toxicity to the biomass. Subsequently, bacteria adaptation to the undiluted influent allowed increasing the nitrogen removal efficiency up to 60–70%. Quasi-stable conditions were achieved after about 110 days. In the second start-up, a quicker stabilization procedure (about 30 days) was demonstrated, with maximum nitrogen removal efficiency between 70 and 80%. In both periods, the compliance with discharge in sewer system (100 mgN/L) was successfully demonstrated. While the special authorization on TN sewer discharge limits is expected to be revoked in the near future, the PN/anammox process represents a solution of outstanding importance to simultaneously preserve environmental, social and economic aspects in the textile district. Different technological solutions for decentralized treatment of DTP wastewater have been evaluated within the EU’s LIFE DeNTreat project, although such preliminary assessment evidenced the absence of competitive alternatives in terms of economic sustainability, thus resulting in more significant impacts on the production sector.

A simplified model for involved biological processes based on mass balance for nitrogen species and COD has been created. This model considered the stoichiometry of AOB, NOB, anammox and heterotrophic bacteria. These bacterial species may be favoured or inhibited depending on the operating conditions of the PN/anammox laboratory pilot, the characteristics of the feeding wastewater, the nitrogen and the COD loading rates. The fraction of NO_2_-N removed by different biological processes, as predicted by the simplified model, is shown in Fig. S1. At the beginning of the experimental activity on WW 1, where diluted wastewater was treated (until day 52), DO level in the reactor could hardly get values lower than 1 mg/L, possibly due to low nitrogen and COD loads. These operating conditions, at a pH of 7.2–7.6, promoted NOB activity, as demonstrated by NO_3_-N concentration up to 47 mg/L at the discharge. During this period, the model predicted that NO_2_-N removal was partly done by NOB (from about 10 to 90%), partly by anammox (up to about 75%) and partly by denitrification (up to about 75%). After this initial phase, when concentrated WW 1 has been treated, NOB activity was probably inhibited by lower DO level in the reactor (0.2–0.4 mg/L). During this period, NO_2_-N has been removed approximately at 50% by anammox process and at 50% by denitrification on average. Therefore, although the presented simplified model represents a preliminary description of involved biological processes, it is worth noticing the importance of NO_2_-N denitrification in the PN/anammox laboratory pilot.

**Fig. 3:**
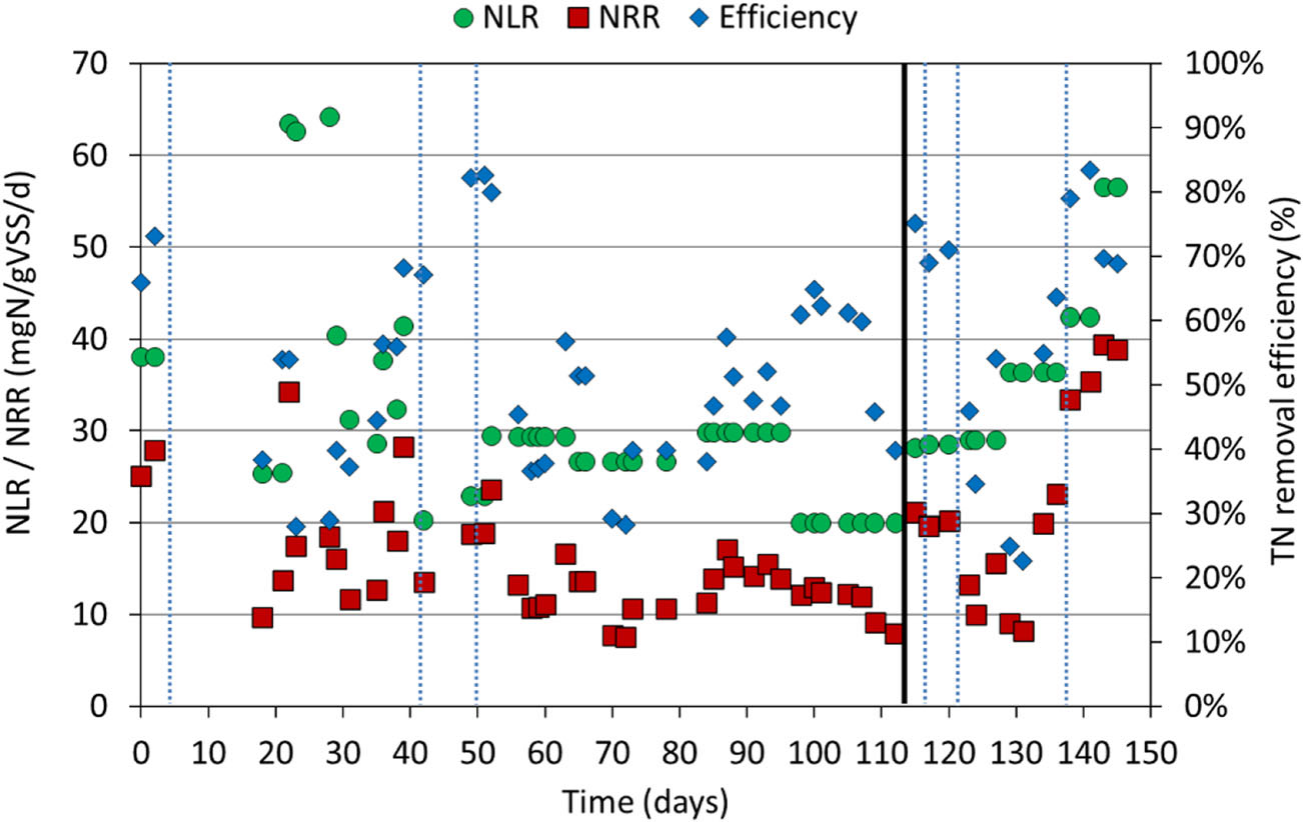
Nitrogen loading rate (NLR), nitrogen removal rate (NRR) and TN removal efficiency achieved in the PN/anammox reactor for experimental activity conducted on WW 1

Figure 3 shows the nitrogen loading rate (NLR in mgN/gVSS/day), the nitrogen removal rate (NRR in mgN/gVSS/day) and the nitrogen removal efficiency (%) achieved with WW 1 in the PN/anammox reactor, indicating about 40 mgN/gVSS/day as maximum value for NRR.

### Microbial composition of the granular biomass

**Fig. 4:**
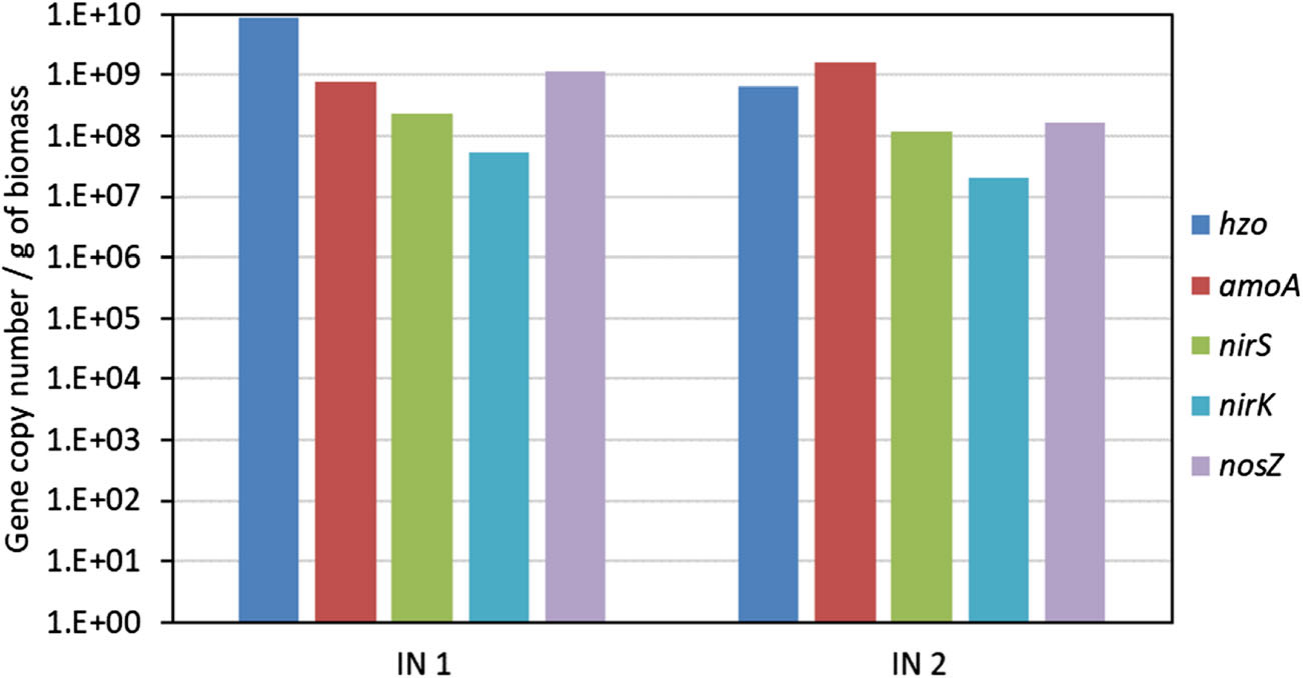
Concentration of the gene copies of anammox bacteria (*hzo*), ammonia oxidizing bacteria (*amoA*) and denitrifying bacteria (*nirS, nirK* and *nosZ*) detected by qPCR in IN 1 and IN 2

The abundance of the main genes involved in nitrogen cycling in the granular biomass batches was assessed by quantitative PCR. Figure 4 shows the concentration of genes related to anammox (*hzo*), AOB (*amoA*) and denitrifiers (*nirS, nirK, nosZ*) in the inocula. The gene copy number of anammox bacteria in IN 1 was one order of magnitude higher than in IN 2. On the contrary, a higher abundance of AOB was detected in IN 2 with respect to IN 1. A conspicuous number of denitrifiers were detected in both biomasses.

**Fig. 5:**
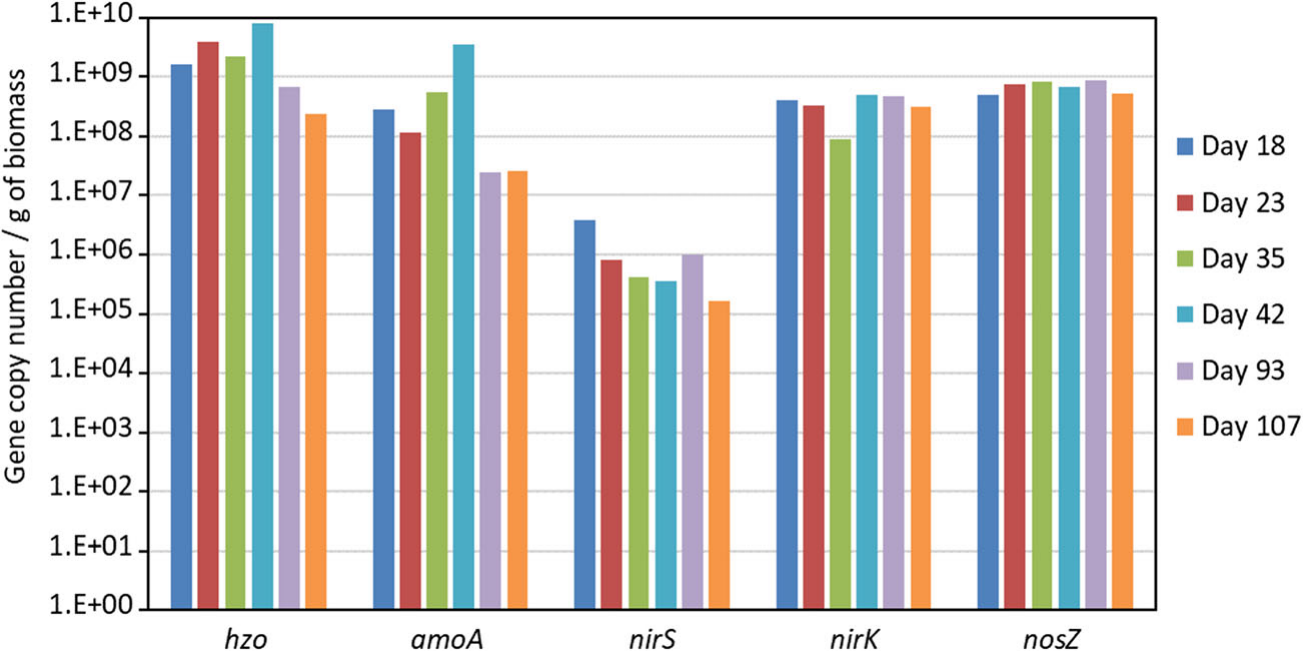
Concentration of the gene copy number of anammox bacteria (*hzo*), ammonia oxidizing bacteria (*amoA*) and denitrifying bacteria (*nirS, nirK* and *nosZ*) detected by qPCR in IN 1 at different days of the experimental activity on WW 1

The evolution of different populations within the first part of the experimental activity on WW 1 is reported in Fig. 5. An increase of anammox and AOB bacteria was observed between day 23 and 42, when the reactors were fed with a mixture of WW 1 (30%) and synthetic wastewater (70%). These findings are in line with the nitrogen removal rate of the reactors, confirming their pivotal role in the process and the requirement of an acclimation phase to the reactor conditions. However, a drastic decrease in anammox and AOB abundances was detected at the end of the period for undiluted wastewater. It is possible that the higher concentration of toxic compounds in the feeding wastewater severely impaired the growth of these bacteria, thereby affecting reactor performance. The abundance of total denitrifiers (*nosZ*) was quite stable over the entire period; gene copy numbers ranged between 8.74 × 10^7^ and 1.17 × 10^9^. Nevertheless, the abundance of *nirS*-type denitrifiers decreased during the period independently on the percentages of WW 1 in the feeding solution. This behaviour may be ascribed to the higher DO level and higher NO_3_-N concentration in the reactor when diluted wastewater was treated.

### Wastewater from other industrial plants

**Fig. 6:**
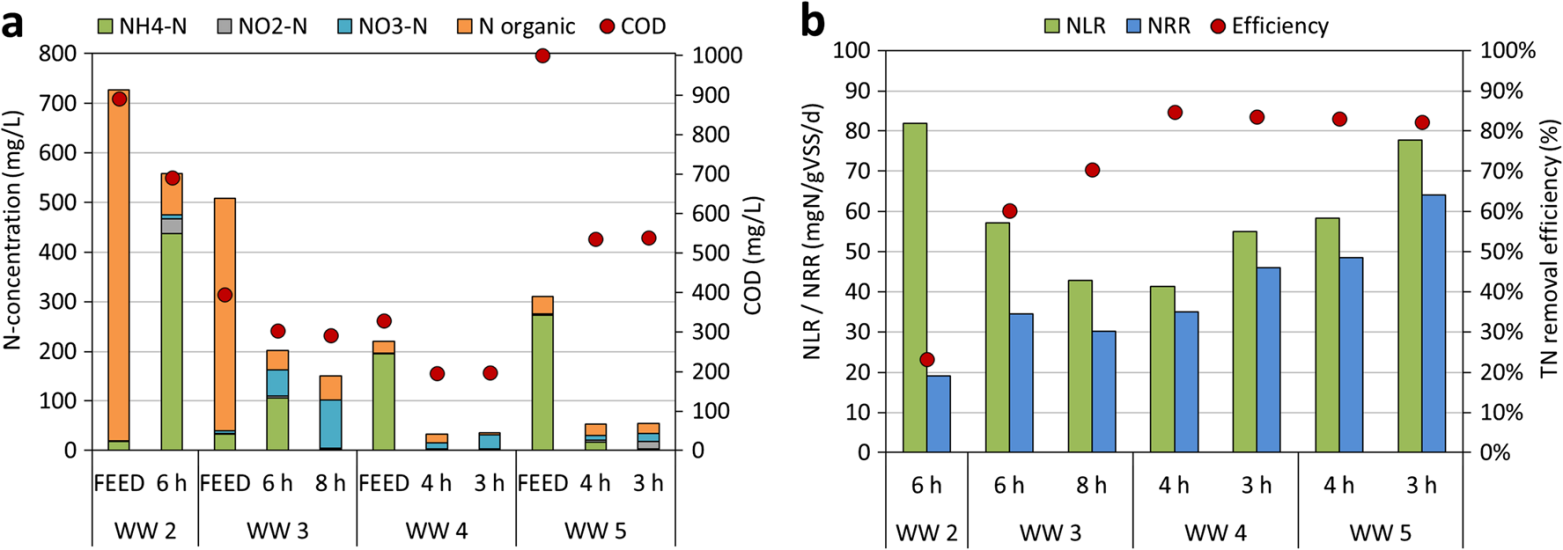
**a** Composition of the PN/anammox reactor influent and effluent and **b** related NLR, NRR and nitrogen removal efficiency achieved in the PN/anammox reactor for experimental activity conducted on wastewater from other industrial plants. Cycle duration is reported

The composition of the PN/anammox reactor influent and effluent for experimental activity conducted on wastewater from other industrial plants is shown in Fig. 6a. For sake of simplicity, only experimental results referring to undiluted samples are reported.

Considering WW 2, despite low TN removal, it is important to notice that a very high ammonification occurred and most of the organic nitrogen was converted into ammoniacal nitrogen. Indeed, bacterial populations in the reactor were able to convert urea into ammonium and also a large proportion of the ammonia was removed via PN/anammox process and not via nitrification/denitrification. Experimental results suggested that nitrogen removal could have been increased by extending the duration of each cycle, in order to enhance PN by AOB bacteria.

Similar results for ammonification have been achieved with WW 3, where the bacterial consortium was able to convert organic nitrogen into ammonium. In this case, 60% of TN was removed through the PN/anammox process. Since high concentration of ammonia (about 100 mg/L) was present in the effluent at the end of the 6 h-cycle, the duration of each cycle was increased up to 8 h. The results showed that about 90% of the organic nitrogen was ammonified and almost 100% of the ammonia was removed. A maximum removal efficiency of 70% was achieved for TN.

On the other hand, the organic fraction in WW 4 and 5 was already converted to ammonia before the PN/anammox process. The experiments allowed to remove 82–85% of TN at the discharge, independently from the duration of the cycles, with average effluent concentrations of 34 and 54 mg/L for WW 4 and WW 5, respectively. These results showed that the nitrogen load might be furtherly increased, with shorter cycles, without any strong effect on TN removal efficiencies.

Figure 6b shows the NLR and the NRR of wastewater from other industrial plants. Nitrogen removal efficiency was higher than 80% for WW 4 and 5, while it was limited to 23% for WW 2, probably due to the too short cycle duration. Moreover, NLR values were in the range 41–55 mgN/gVSS/day and 58–78 mgN/gVSS/day for WW 4 and 5, respectively, and may have been increased further. In conclusion, it was demonstrated that WW 4 and 5 can be treated by the PN/anammox process with high efficiencies. Conversely, WW 2 and 3 needed longer cycles duration and lower nitrogen loads to be treated, possibly favouring the conversion of organic nitrogen to ammoniacal nitrogen.

### Process modelling

**Fig. 7:**
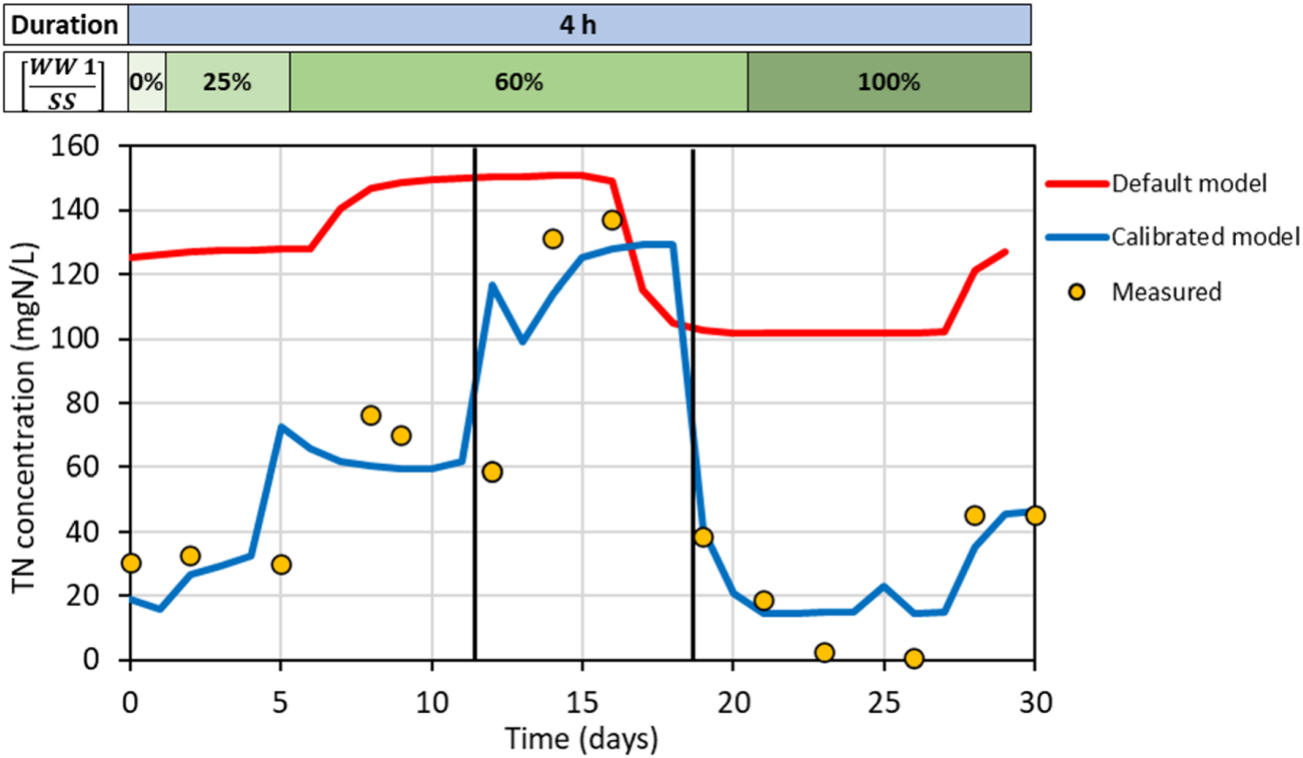
Experimental and modelling (for default and calibrated parameters) data referred to the last period of experimental activity on WW 1. Wastewater dilution ratio and changes in WW 1 batches (black lines) are reported

Model calibration led to the accurate description of experimental data obtained during the last period of experimental activity on WW 1, as evidenced in Fig. 7. In order to improve model accuracy, the modification in the composition of wastewater during the modelled experimental activity was considered. In fact, three different batches of WW 1 were used for reactor feeding during the modelled period of operation. In conclusion, wastewater characterization, including a proper fractionation (nitrogen species, COD), and a correct setting of modelling kinetic parameters are important factors in achieving an accurate modelling of experimental data.

**Fig. 8:**
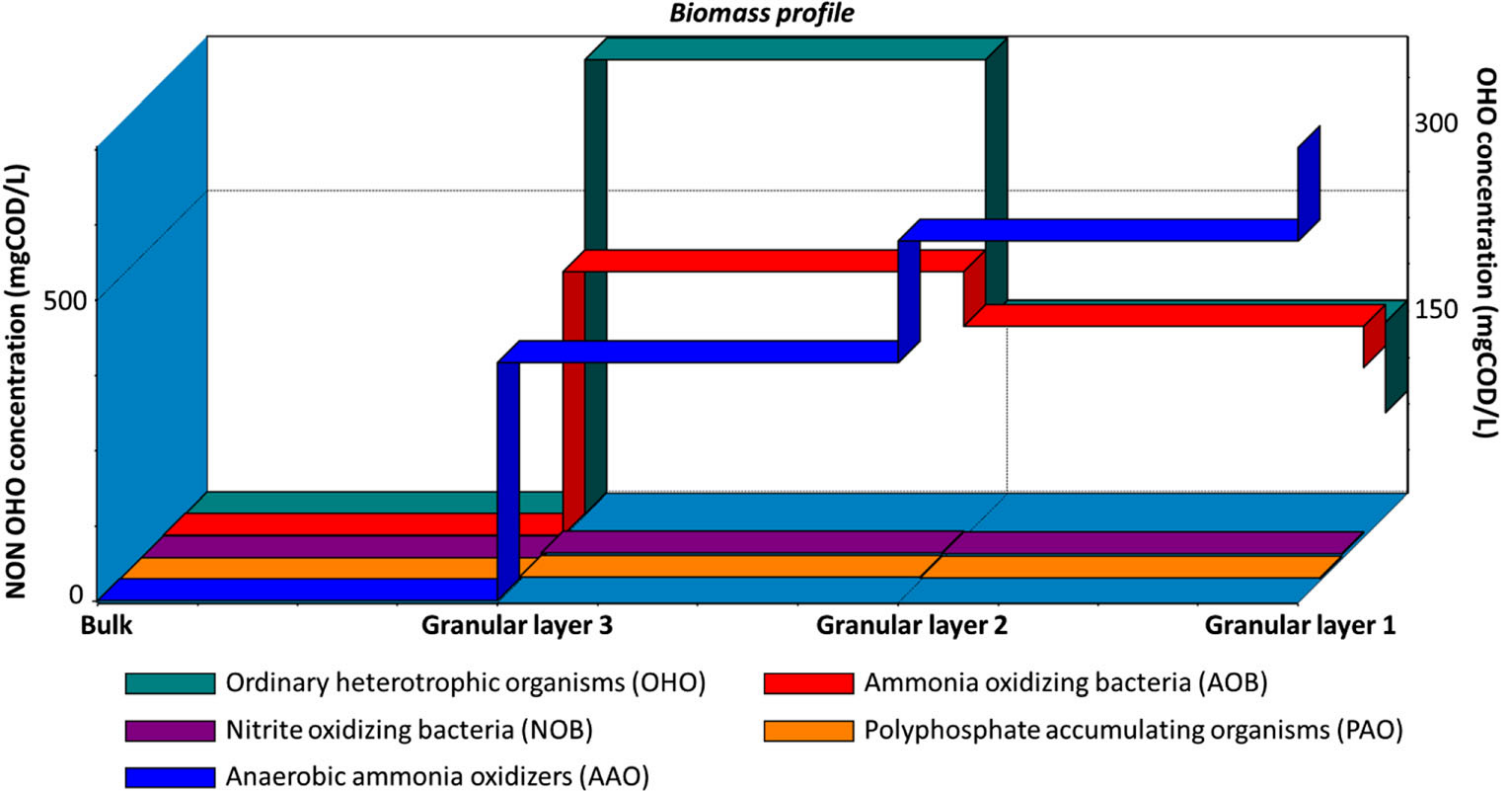
Simulated concentration profile of bacteria species under hypothetical steady state

Once the model was developed, it was possible to simulate reactor start-up until steady state. According to model estimation, at least 65 days are required before reaching removal efficiencies of approximately 75% on TN concentrations at the discharge, in order to obtain values of about 50 mg/L, in compliance with limits for the discharge into the sewer. It is important to note that the results on TN removal efficiencies generated by dynamic simulations depended on the relative abundance of the different bacterial species inside the granules predicted by the model. In particular, the modelling tool allowed to simulate the composition profile of the biofilm during the periods of operation of the reactor. In this case, upon reaching steady state, the model assumed a predominance of heterotrophic bacteria (300 mgCOD/L) in the external layer of the biofilm, while the concentration of anammox bacteria increased in the deeper layers, up to values of 450 mgCOD/L, as illustrated in Fig. 8. These results were probably determined by the extremely low concentration of DO expected in the deeper layers of the biofilm, resulting from diffusion limitations, which could favour the anammox bacteria.

### Process sustainability assessment

Although the PN/anammox process could lower TN content below the regulatory limit for discharge into the sewer, some other KPIs are essential in determining process sustainability.

The energy consumption expected by the model was reduced from 1,026,600 kWh/year for scenario 0 to 874,500 kWh/year for scenario 1, which consisted approximately in a 15% reduction, considering the complete treatment of DTP wastewater discharged in the sewer system.

Regarding GHG emissions, the analysis was limited to the emission of nitrous oxide (N_2_O), GHGs with a global warming potential of 298 gCO_2_-eq/gN_2_O, based on a time horizon of 100 years (Forster et al. 2007). Based on model estimation, the PN/anammox reactor resulted in an emission of approximately 0.045 kgN_2_O/day, corresponding to an annual emission of approximately 16 kgN_2_O in case that the PN/anammox is applied in scenario 1. However, it is necessary to emphasize that the N_2_O emissions generated by the PN/anammox process are still under investigation and that emission coefficients are affected by significant uncertainties. Literature reports different ratios between the mass of N_2_O emitted and the total mass of nitrogen at the feed, including 0.4–2% (Law et al. 2012; Castro-Barros et al. 2015) and 0.2–0.5% (Jia et al. 2018). These differences are attributable to the important influence on the emissions of the operating conditions (COD/N ratio, reactor configuration, input nitrogen load, DO concentration, type of process control, adaptation of biomass, composition of the microbial consortium…). Considering the centralized WWTP in two scenarios, the model allowed to estimate emission factors reduced from 0.24% ± 0.25% to 0.11% ± 0.25% switching from scenario 0 to scenario 1. These values are confirmed in literature from the work of Baresel et al. (2016) conducted on a conventional activated WWTP and 50% lower than those reported by Peu et al. (2006) for a similar plant operated with alternating aeration cycles.

At the moment, the efficient operation of the centralized WWTP requires the addition of external carbon for completing the denitrification process. The implementation of scenario 1, resulting in the reduction of nitrogen load entering WWTP and the consequent modification of the COD/N ratio up to values of 12, could result in avoiding the dosage external carbon, resulting in an economic saving of about 70,000 €/year.

The quantity of sludge produced is reduced from 558 kgTSS/day for scenario 0 to 418 kgTSS/day for scenario 1, thus indicating a 25% reduction.

In conclusion, KPIs estimated from model-based simulations show a substantial modification of the impacts related to the treatment of wastewater in the two scenarios: the on-site treatment prior to the final step in a centralized WWTP allowed reductions in energy consumption (− 15%), reduction in GHGs emission, elimination of external carbon source at existing WWTP and a reduced sludge production (− 25%).

## Conclusions

The research work assessed the application of PN/anammox process in a single-stage granular SBR for on-site decentralized treatment of DTP wastewater. Process feasibility was demonstrated, since the process was able to lower TN concentration in the effluent down to 50 mg/L, below limits on discharge in the sewer system. A process stabilization strategy was reported, highlighting the importance of accurate control of operating conditions, especially during the initial acclimation period. Significant reversible reductions in bacterial activity were observed when industrial textile wastewater ratio was increased, probably due to wastewater toxicity, and the presence of specific consortia in the biomass capable of converting urea to ammoniacal nitrogen was noticed. Process modelling was applied to experimental data and a good fitting was obtained, indicating the importance of a case-specific calibration of modelling parameters. In conclusion, KPIs estimated a substantial modification of the impacts related to wastewater treatment in the two scenarios, indicating the decentralized treatment of nitrogen-rich industrial effluents as an effective solution. As a further step, the EU’s LIFE DeNTreat project aims at demonstrating the feasibility in treating wastewaters discharged from DTP companies by means of a full scale PN/anammox pilot plant.

## Electronic supplementary material

ESM 1 (DOCX 146 kb)

## Data Availability

All data generated or analysed during this study are included in this published article and its supplementary information files.

## References

[CR1] Baresel C, Andersson S, Yang J, Andersen MH (2016). Comparison of nitrous oxide (N2O) emissions calculations at a Swedish wastewater treatment plant based on water concentrations versus off-gas concentrations. Adv Clim Chang Res.

[CR2] Barker PS, Dold PL (1997). General model for biological nutrient removal activated-sludge systems: model presentation. Water Environ Res.

[CR3] Canziani R, Emondi V, Garavaglia M, Malpei F, Pasinetti E, Buttiglieri G (2006). Effect of oxygen concentration on biological nitrification and microbial kinetics in a cross-flow membrane bioreactor (MBR) and moving-bed biofilm reactor (MBBR) treating old landfill leachate. J Membr Sci.

[CR4] Carvajal-Arroyo JM, Puyol D, Li G, Sierra-Álvarez R, Field JA (2014). The role of pH on the resistance of resting- and active anammox bacteria to NO2 - inhibition. Biotechnol Bioeng.

[CR5] Castro-Barros CM, Daelman MRJ, Mampaey KE, van Loosdrecht MCM, Volcke EIP (2015). Effect of aeration regime on N2O emission from partial nitritation-anammox in a full-scale granular sludge reactor. Water Res.

[CR6] Cho S, Kambey C, Nguyen VK (2020) Performance of anammox processes for wastewater treatment: a critical review on effects of operational conditions and environmental stresses. Water (Switzerland) 12. 10.3390/w12010020

[CR7] Egli K, Fanger U, Alvarez PJJ, Siegrist H, van der Meer JR, Zehnder AJB (2001). Enrichment and characterization of an anammox bacterium from a rotating biological contactor treating ammonium-rich leachate. Arch Microbiol.

[CR8] Forster P, Ramaswamy V, Artaxo P, Nakajima T, Ramanathan V (2007). Changes in atmospheric constituents and in radiative forcing. Climate change 2007: the physical science basis.

[CR9] Global Industry Analysts Inc. (2018) (MCP-6171) Textile printing – Market analysis, trends, and forecasts.https://www.strategyr.com/market-reporttextile-printing-forecasts-global-industry-analysts-inc.asp. Accessed 28 Sept 2020

[CR10] Gonzalez-Martinez A, Muñoz-Palazon B, Rodriguez-Sanchez A, Gonzalez-Lopez J (2018). New concepts in anammox processes for wastewater nitrogen removal: recent advances and future prospects. FEMS Microbiol Lett.

[CR11] Gonzalez-Silva BM, Rønning AJ, Andreassen IK, Bakke I, Cervantes FJ, Østgaard K, Vadstein O (2017). Changes in the microbial community of an anammox consortium during adaptation to marine conditions revealed by 454 pyrosequencing. Appl Microbiol Biotechnol.

[CR12] Hu Z, Lotti T, van Loosdrecht M, Kartal B (2013). Nitrogen removal with the anaerobic ammonium oxidation process. Biotechnol Lett.

[CR13] Jetten MSM, Strous M, van de Pas-Schoonen KT, Schalk J, van Dongen UGJM, van de Graaf AA, Logemann S, Muyzer G, van Loosdrecht MCM, Kuenen JG (1998). The anaerobic oxidation of ammonium. FEMS Microbiol Rev.

[CR14] Jia M, Castro-Barros CM, Winkler MKH, Volcke EIP (2018). Effect of organic matter on the performance and N2O emission of a granular sludge anammox reactor. Environ Sci Water Res Technol.

[CR15] Jin RC, Yang GF, Yu JJ, Zheng P (2012). The inhibition of the anammox process: a review. Chem Eng J.

[CR16] Jubany I, Lafuente J, Baeza JA, Carrera J (2009). Total and stable washout of nitrite oxidizing bacteria from a nitrifying continuous activated sludge system using automatic control based on oxygen uptake rate measurements. Water Res.

[CR17] Lackner S, Gilbert EM, Vlaeminck SE, Joss A, Horn H, van Loosdrecht MCM (2014). Full-scale partial nitritation/anammox experiences – an application survey. Water Res.

[CR18] Law Y, Ye L, Pan Y, Yuan Z (2012). Nitrous oxide emissions from wastewater treatment processes. Philos Trans R Soc B Biol Sci.

[CR19] Li J, Li J, Gao R, Wang M, Yang L, Wang X, Zhang L, Peng Y (2018). A critical review of one-stage anammox processes for treating industrial wastewater: optimization strategies based on key functional microorganisms. Bioresour Technol.

[CR20] Lotti T, Kleerebezem R, Hu Z, Kartal B, de Kreuk MK, van Erp Taalman Kip C, Kruit J, Hendrickx TLG, van Loosdrecht MCM (2015). Pilot-scale evaluation of anammox-based mainstream nitrogen removal from municipal wastewater. Environ Technol.

[CR21] Lotti T, Scaglione D, Teli A (2014). Rimozione completamente autotrofa dell’ azoto con batteri anammox: passato, presente e futuro. Ing dell’Ambiente.

[CR22] Mulder A (2003). The quest for sustainable nitrogen removal technologies. Water Sci Technol.

[CR23] Peu P, Béline F, Picard S, Héduit A (2006) Measurement and quantification of nitrous oxide emissions from municipal activated sludge plants in France. Proceeding of 5th IWA World Water Congress. Beijing, China

[CR24] Puyol D, Carvajal-Arroyo JM, Li GB, Dougless A, Fuentes-Velasco M, Sierra-Alvarez R, Field JA (2014). High pH (and not free ammonia) is responsible for anammox inhibition in mildly alkaline solutions with excess of ammonium. Biotechnol Lett.

[CR25] Rice EW, Baird RB, Eaton AD (2017) Standard methods for the examination of water and wastewater. American Public Health Association, American Water Works Association, Water Environment Federation

[CR26] Scaglione D, Lotti T, Menin G (2016). Complete autotrophic process for nitrogen removal from ink- jet printing wastewater. Chem Eng Trans.

[CR27] Siegrist H, Salzgeber D, Eugster J, Joss A (2008). Anammox brings WWTP closer to energy autarky due to increased biogas production and reduced aeration energy for N-removal. Water Sci Technol.

[CR28] Strous M, Kuenen JG, Jetten MSM (1999) Key physiology of anaerobic ammonium oxidation. Appl Environ Microbiol 65:3248–3250. 10.1128/AEM.65.7.3248-3250.1999 10.1128/aem.65.7.3248-3250.1999 PMC91484 10388731

[CR29] Strous M, Van Gerven E, Kuenen JG, Jetten M (1997). Effects of aerobic and microaerobic conditions on anaerobic ammonium-oxidizing (anammox) sludge. Appl Environ Microbiol.

[CR30] Takács I, Bye CM, Chapman K, Dold PL, Fairlamb PM, Jones RM (2007). A biofilm model for engineering design. Water Sci Technol.

[CR31] van Dongen U, Jetten MSM, van Loosdrecht MCM (2001). The SHARON®-Anammox® process for treatment of ammonium rich wastewater. Water Sci Technol.

[CR32] Van Hulle SW, Volcke EI, Teruel JL (2007). Influence of temperature and pH on the kinetics of the Sharon nitritation process. J Chem Technol Biotechnol.

[CR33] Yang Y, Zuo J-E, Shen P, Gu X-S (2006). Influence of temperature, pH value and organic substance on activity of ANAMMOX sludge. Huanjing Kexue/Environ Sci.

[CR34] Yoo H, Ahn K-H, Lee H-J, Lee KH, Kwak YJ, Song KG (1999). Nitrogen removal from synthetic wastewater by simultaneous nitrification and denitrification (SND) via nitrite in an intermittently-aerated reactor. Water Res.

