# Supplementary material for: Autotrophic nitrogen removal for decentralized treatment of ammonia-rich industrial textile wastewater: process assessment, stabilization and modelling
Source: Environ Sci Pollut Res Int. 2020 Oct 20;28(34):46643–54. doi: 10.1007/s11356-020-11231-y (PMC8384811; doi:10.1007/s11356-020-11231-y)
Supplement: Supplementary file 1 — (DOCX 146 kb) [file 11356_2020_11231_MOESM1_ESM.docx]

**Autotrophic nitrogen removal for decentralized treatment of ammonia-rich industrial textile wastewater: process assessment, stabilization and modelling**

Simone Visigalli^1^, Andrea Turolla^1^, Giacomo Bellandi^2^, Micol Bellucci^1^, Elisa Clagnan^3^, Lorenzo Brusetti^3^, Mingsheng Jia^1^, Roberto Di Cosmo^4^, Glauco Menin^1^, Martina Bargna^5^, Giovanni Bergna^5^, Roberto Canziani^1^ *

^1^ Department of Civil and Environmental engineering – Environmental section, Politecnico di Milano, Piazza Leonardo da Vinci 32, 20133 Milano, Italy

^2^ AM-TEAM, Oktrooiplein 1, 9000 Ghent, Belgium

^3^ Faculty of Science and Technology, Free University of Bolzano, Piazza Università 1, 39100 Bolzano, Italy

^4^ Gruppo CAP, Via del Mulino 2, 20090 Assago, Italy

^5^ Lariana Depur, Via Laghetto 1, 22073 Fino Mornasco, Italy

* Corresponding Author: **Roberto Canziani**

Telephone: (+39) 02 2399 6410

E-mail: [roberto.canziani@polimi.it](mailto:roberto.canziani@polimi.it)

Table S1 Composition of synthetic wastewater and of trace element solution.

|  | Compound | Concentration (g/L) |
| --- | --- | --- |
| Synthetic solution | NH_4_Cl | 0.573 |
|  | CaCl_2_⋅2H_2_O | 0.075 |
|  | MgSO_4_⋅7H_2_O | 0.101 |
|  | KH_2_PO_4_ | 0.340 |
|  | K_2_HPO_4_ | 0.436 |
|  | FeSO_4_⋅5H_2_O | 0.019 |
|  | Trace elements solution | 1.25 mL/L |
| Trace elements solution | EDTA | 15.0 |
|  | ZnSO_4_·7H_2_O | 0.43 |
|  | CoCl_2_·6H_2_O | 0.24 |
|  | MnCl_2_·4H_2_O | 1.00 |
|  | CuSO_4_·5H_2_O | 0.25 |
|  | (NH_4_)_6_Mo_7_O_24_·4H_2_O | 0.22 |
|  | NiCl_2_·6H_2_O | 0.20 |
|  | NaSeO_4_·10H_2_O | 0.20 |
|  | H_3_BO_3_ | 0.014 |

Table S2 Duration of the different phases used in the experiments with the five wastewaters.

| Cycle duration (h) | Feed  (min) | Reaction (min) | Settling (min) | Discharge (min) |
| --- | --- | --- | --- | --- |
| 3 | 106.5 | 61.9 | 1.0 | 0.5 |
| 4 | 106.5 | 121.9 | 1.0 | 0.5 |
| 6 | 181.8 | 176.6 | 1.0 | 0.5 |
| 8 | 181.8 | 296.6 | 1.0 | 0.5 |

Table S3 Summary of the gene targets, primer sets and PCR conditions.

| **Genes** | **Pathways** | **Reaction** | **Primers** | **Amplicon size (bp)** | **qPCR condition** | **Reference** |
| --- | --- | --- | --- | --- | --- | --- |
| *hzo* | Anammox |  | hzoF - hzoR1 | 740 | 95 ˚C-10 m; 40 cycles: 95 ˚C-30 s, 56 ˚C-20 s, acquisition at 72 ˚C-40 s; 95 ˚C-15 s; dissociation curve. (Kong et al. 2013) | (Kong et al. 2013) |
| *amoA* | Nitrification (AOB) | Ammonium oxidation to hydroxylamine | amoA-1F - amoA-2R | 491 | 95 ˚C-10 m; 45 cycles: 95 ˚C-1 m, 54 ˚C-1 m, acquisition at 72 ˚C-1 m; 72 ˚C-10 m; dissociation curve. (Segal et al. 2017) | (Rotthauwe et al. 1997) |
| *nirK* | Denitrification | Nitrite reduction to nitric oxide | nirK876 - nirK1040 | 164 | 95 ˚C-15 min; 6 cycles: 95 ˚C-15 s, 63 to 58 ˚C-30 s with a decrease of 1 ˚C every cycle, 72 ˚C-30 s, 80 ˚C-15 s; 40 cycles: 95 ˚C-15 s, 60 ˚C-30 s, 72 ˚C-30 s, acquisition at 80 ˚C-30 sec; 95 ˚C-15 sec; dissociation curve. (Henry et al. 2004) | (Hallin et al. 2009) |
| *nirS* | Denitrification | Nitrite reduction to nitric oxide | Cd3aF - R3cd | 416 | 95 ˚C-10 m; 40 cycles: 95 ˚C-30 s, 57 ˚C-20 s, acquisition at 72 ˚C-30 s; 95 ˚C-15 s; dissociation curve. (Thompson et al. 2016) | (Michotey et al. 2000; Throback et al. 2004) |
| *nosZ* | Denitrification | Nitrous oxide reduction to dinitrogen | nosZ2F - nosZ2R | 267 | 95 ˚C-15 m; 6 cycles: 95 ˚C-15 s, 65 to 60 ˚C-30 s with a decrease of 1˚C every cycle, 72 ˚C-30 s, 80 ˚C-15 s; 40 cycles: 95 ˚C-15 s, 60 ˚C-15 s, 72 ˚C-30 s, acquisition at 80 ˚C-30 s; 95 ˚C-15 s; dissociation curve. (Henry et al. 2006) | (Henry et al. 2006) |

Table S4 Sizing parameters for PN/anammox process model.

| Parameter | Value |
| --- | --- |
| Reactor volume (m^3^) | 0.002 |
| Depth (m) | 0.5 |
| Thickness (m) | 0.1 |
| N° diffusers | 8 |
| Diffusers diameter (mm) | 270 |
| Diffusers area (m^2^) | 0.041 |
| Max air flux (m^3^/h) | 10 |
| Decay level (%) | 96.5 |
| DO set point (mg/L) | 0.1 |
| N° of internal layers | 3 |
| Granules diameter (mm) | 3 |

Table S5 Kinetic parameters of bacteria species.

| Parameter | Default | Used |
| --- | --- | --- |
| AOB |  |  |
| Maximum specific growth rate [1/d] | 0.9 | 0.9 |
| Substrate (NH_4_) half-saturation [mgN/L] | 0.7 | 0.7 |
| AOB denitrification DO half-saturation [mg/L] | 0.1 | 0.1 |
| AOB denitrification HNO_2_ half-saturation [mgN/L] | 5.00E-06 | 5.00E-06 |
| Aerobic decay rate [1/d] | 0.17 | 0.17 |
| Anoxic/anaerobic decay rate [1/d] | 0.08 | 0.08 |
| KiHNO_2_ [mmol/L] | 0.005 | 0.005 |
| NOB |  |  |
| Maximum specific growth rate [1/d] | 0.7 | 0.4 |
| Substrate (NO_2_) half-saturation [mgN/L] | 0.1 | 0.1 |
| Aerobic decay rate [1/d] | 0.17 | 0.21 |
| Anoxic/anaerobic decay rate [1/d] | 0.08 | 0.08 |
| KiNH_3_ [mmol/L] | 0.075 | 0.075 |
| Anammox |  |  |
| Maximum specific growth rate [1/d] | 0.2 | 0.25 |
| Substrate (NH_4_) half-saturation [mgN/L] | 2 | 2 |
| Substrate (NO_2_) half-saturation [mgN/L] | 1 | 1 |
| Aerobic decay rate [1/d] | 0.019 | 0.019 |
| Anoxic/anaerobic decay rate [1/d] | 0.0095 | 0.0095 |
| OHO |  |  |
| Maximum specific growth rate [1/d] | 3.2 | 0.01 |
| Substrate half-saturation [mgCOD/L] | 5 | 5 |
| Anoxic growth factor [-] | 0.5 | 0.5 |
| Denitrification N_2_ production (NO_3_ or NO_2_) [-] | 0.5 | 0.5 |
| Aerobic decay rate [1/d] | 0.62 | 0.62 |
| Anoxic decay rate [1/d] | 0.233 | 0.233 |
| Anaerobic decay rate [1/d] | 0.131 | 0.131 |


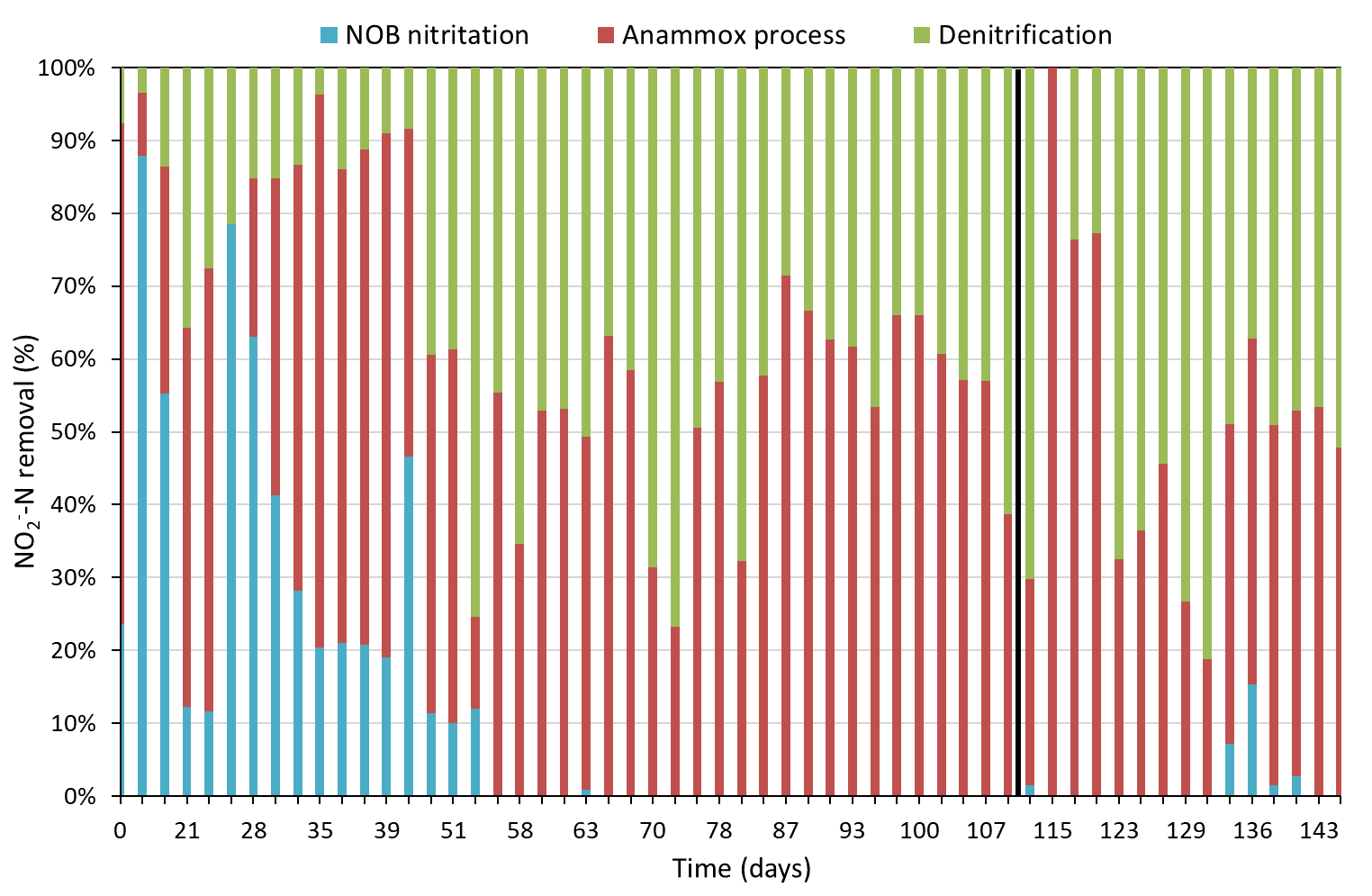
Figure S1 NO_2_-N removal by different biological processes (NOB nitritation, anammox process and denitrification) during experimental activity on WW 1.

**References**

Hallin S, Jones CM, Schloter M, Philippot L (2009) Relationship between N-cycling communities and ecosystem functioning in a 50-year-old fertilization experiment. ISME J 3:597–605. <https://doi.org/10.1038/ismej.2008.128>

Henry S, Baudoin E, López-Gutiérrez JC, Martin-Laurent F, Brauman A, Philippot L (2004) Quantification of denitrifying bacteria in soils by nirK gene targeted realtimePCR. J Microbiol Methods 59:327–335. <https://doi.org/10.1016/j.mimet.2004.07.002>

Henry S, Bru D, Stres B, Hallet S, Philippot L (2006) Quantitative detection of the nosZ gene, encoding nitrous oxide reductase, and comparison of the abundances of 16S rRNA, narG, nirK, and nosZ genes in soils. Appl Environ Microbiol 72:5181–5189. <https://doi.org/10.1128/AEM.00231-06>

Kong L, Jing H, Kataoka T, Buchwald C, Liu H (2013) Diversity and spatial distribution of hydrazine oxidoreductase (hzo) gene in the oxygen minimum zone off Costa Rica. PLoS One 8:e78275. <https://doi.org/10.1371/journal.pone.0078275>

Michotey V, Méjean V, Bonin P (2000) Comparison of methods for quantification of cytochrome cd1-denitrifying Bacteria in environmental marine samples. Appl Environ Microbiol 66:1564–1571. <https://doi.org/10.1128/AEM.66.4.1564-1571.2000>

Rotthauwe JH, Witzel KP, Liesack W (1997) The ammonia monooxygenase structural gene amoA as a functional marker: molecular fine-scale analysis of natural ammonia-oxidizing populations. Appl Environ Microbiol 63:4704–4712. <https://doi.org/10.1128/AEM.63.12.4704-4712.1997>

Segal LM, Miller DN, McGhee RP et al (2017) Bacterial and archaeal ammonia oxidizers respond differently to long-term tillage and fertilizer management at a continuous maize site. Soil Tillage Res 168:110–117. <https://doi.org/10.1016/j.still.2016.12.014>

Thompson KA, Bent E, Abalos D, Wagner-Riddle C, Dunfield KE (2016) Soil microbial communities as potential regulators of in situ N2O fluxes in annual and perennial cropping systems. Soil Biol Biochem 103:262–273. <https://doi.org/10.1016/j.soilbio.2016.08.030>

Throback IN, Enwall K, Jarvis A, Hallin S (2004) Reassessing PCR primers targeting nirS, nirK and nosZ genes for community surveys of denitrifying bacteria with DGGE. FEMS Microbiol Ecol 49:401–417. https://doi.org/10.1016/j.femsec.2004.04.011
